# Greater deformation and lower stiffness, but similar ultimate failure strength with fixed‐loop cortical suspensory device compared to metal interference screw for femoral fixation of bone–patellar tendon–bone grafts in anterior cruciate ligament reconstruction

**DOI:** 10.1002/jeo2.70921

**Published:** 2026-09-22

**Authors:** Alexander Nagel Tandberg, Marek Zegzdryn, Jan‐Egil Brattgjerd, Gilbert Moatshe, Arne Kristian Aune

**Affiliations:** ^1^ Department of Orthopedic Surgery Martina Hansens Hospital Bærum Norway; ^2^ University of Oslo, UIO Oslo Norway; ^3^ Department of Orthopedic Surgery Sørlandet Hospital Arendal Norway; ^4^ Department of Orthopedic Surgery Oslo University Hospital, Ullevål Oslo Norway; ^5^ Aleris Hospital Drammen Norway

**Keywords:** anterior cruciate ligament reconstruction, biomechanical testing, bone–patellar tendon–bone graft, cortical suspensory device, interference screw

## Abstract

**Purpose:**

The purpose of this study was to compare a contemporary fixed‐loop cortical suspensory devices (CSDs) with metal interference screws (IS) for femoral bone–patellar tendon–bone (BPTB) graft fixation in anterior cruciate ligament reconstruction (ACLR).

**Methods:**

Seven matched pairs of fresh‐frozen human cadaveric knees underwent anatomical ACLR with BPTB grafts. Femoral fixation was randomized to a fixed‐loop CSD or metal IS. Specimens underwent cyclic loading at 50 and 100 N at 1 Hz until failure or up to 1000 cycles. Comparison between the fixation methods for cyclic displacement, displacement at failure, construct stiffness and ultimate load to failure was performed. Comparisons between fixation methods were performed using paired *t* tests.

**Results:**

Fixed‐loop CSD fixation demonstrated significantly greater cyclic displacement than IS fixation (2.8 ± 0.9 vs. 1.7 ± 0.5 mm; *p* = 0.008). Displacement at failure was significantly higher (12.3 ± 1.7 vs. 7.9 ± 1.1 mm; *p* = 0.004), and construct stiffness was lower (46 ± 10 vs. 84 ± 18 N/mm; *p* = 0.002) with CSD fixation compared to IS fixation. There was no significant difference in ultimate load to failure (CSD fixation; 563 ± 109 vs. IS fixation; 668 ± 217 N; *p* = 0.31). Failure occurred mainly as lateral cortical fractures with CSD and proximal graft avulsions with IS fixation.

**Conclusion:**

Fixed‐loop CSD fixation of BPTB grafts demonstrated greater deformation and lower stiffness than IS fixation, and with similar ultimate failure strength. The clinical relevance of the observed difference remains uncertain.

**Level of Evidence:**

N/A.

**Abbreviations**
ACLR: anterior cruciate ligament reconstruction
BPTB: bone–patellar tendon–bone
CSD: cortical suspensory devices
IS: interference screws

## INTRODUCTION

Graft selection influences revision risk in anterior cruciate ligament reconstruction (ACLR) [20]. Bone–patellar tendon–bone (BPTB) autografts are associated with lower failure rates than hamstring tendon autografts [12, 16]. Among surgical variables, femoral fixation has been identified as a key modifiable factor influencing graft survival [17, 19, 22].

Because static testing may demonstrate fixation strengths exceeding those required during early rehabilitation [13], dynamic testing has increasingly been incorporated to better reflect clinical loading conditions [2, 14]. Early graft stability before biological incorporation depends on mechanical properties of the fixation construct; therefore, ex vivo biomechanical evaluation remains clinically relevant.

Interference screws (IS) remain a commonly used method for femoral fixation of BPTB grafts [24]. Biomechanically, their effectiveness is attributed to aperture fixation, providing high construct stiffness and ultimate failure strength while minimizing bone–graft motion within the tunnel [3, 15]. Ex vivo failure typically occurs at the fixation interface and includes bone block pull‐out, bone block fracture, and graft avulsion [14].

Cortical suspensory devices (CSDs) are used as an alternative method for femoral fixation in primary ACLR with BPTB grafts [24]. Fixed‐loop designs, in which a cortical button is connected to the graft by a continuous loop, have been reported to provide favourable tunnel healing with reliable bone plug integration [23]. Biomechanically, load transfer with CSD fixation occurs across a longer construct spanning the lateral femoral cortex. Compared with adjustable‐loop devices that rely on locking mechanisms, fixed‐loop constructs demonstrate more consistent displacement patterns, emphasizing the potential importance of loop design [10, 21]. However, although differences in ultimate load to failure and cyclic displacement have been reviewed [8], no corresponding clinical differences have been demonstrated [5]. Ex vivo failure patterns include suture rupture and bone block fracture at the suture hole with suture pull‐out, indicating both device‐ and interface‐related failure mechanisms [3, 7, 14]. Despite high ultimate failure loads, concerns remain regarding reduced construct stiffness and increased cyclic displacement compared with IS fixation [3, 7]. However, earlier biomechanical studies of BPTB fixation primarily evaluated tape‐ or suture‐based button constructs rather than contemporary fixed‐loop designs, which may limit direct applicability to current ACLR, particularly in young patients at risk of revision.

The purpose of this study was to evaluate femoral fixation of BPTB grafts using a fixed‐loop CSD compared with metal IS fixation with respect to biomechanical performance. The hypothesis is that fixed‐loop CSD fixation would demonstrate greater cyclic displacement and lower construct stiffness than IS fixation; however, there will be no difference in ultimate load to failure or ultimate load to failure will be comparable.

## MATERIALS AND METHODS

### Study design

An in vitro biomechanical study was performed and designed as a paired randomized investigation using human cadaveric specimens. Allocation was performed using an online randomization platform prior to specimen preparation. Each pair was tested using two different femoral fixation techniques, allowing within‐pair comparisons. Within each pair, one femur was assigned to CSD fixation and the contralateral femur to IS fixation.

### Specimen preparation

After approval by the Regional Committee for Medical and Health Research Ethics (approval no. 2018/2182), eight matched pairs of fresh‐frozen human cadaveric knees from Caucasian male donors (mean age 41 years, range: 29–49) were obtained (ScienceCare). Specimens from relatively young adult donors were requested in order to reflect the ACLR population. Donors had no known history of prior knee surgery or bone pathology. Specimens were stored at −20°C and thawed overnight at room temperature before preparation. To obtain standardized grafts, the central third of the patellar ligament was harvested. The BPTB grafts included triangular bone blocks from both the distal patella and proximal tibia, measuring approximately 20 mm in length and 10 mm in anterior width each. They were carefully trimmed to pass through a 9‐mm sizing tube. All grafts were wrapped in saline‐soaked gauze and kept at room temperature until fixation. The distal femurs were prepared for biomechanical testing. They were transected 15 cm proximal to the joint line and stripped of all soft tissues. Limited removal of the medial femoral condyle was performed to facilitate testing [18]. All surgical procedures were performed by two experienced orthopaedic surgeons (M.Z. and A.N.T.).

### Fixation techniques

**Figure 1:**
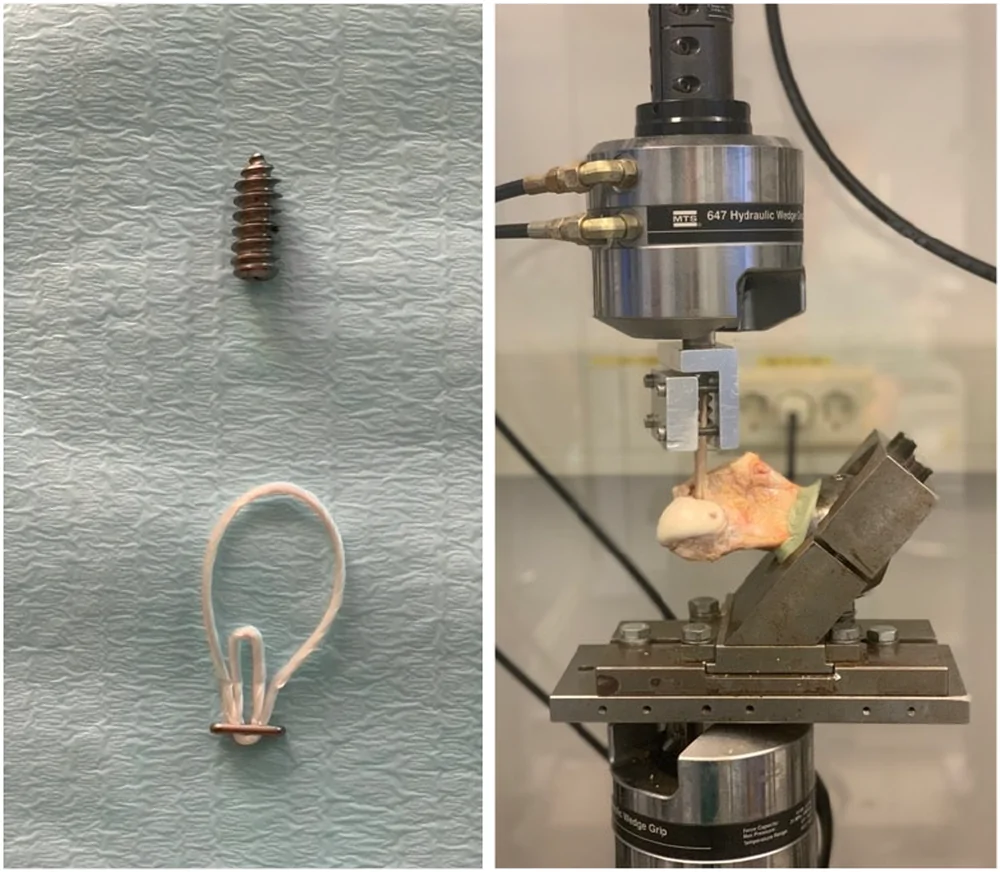
Femoral fixation devices and experimental setup. Left: The femoral fixations used for anterior cruciate ligament reconstruction: Soft Silk 7 × 20 mm interference screw (on top) and Endobutton BTB (below). Right: The experimental set‐up for uniaxial tensile testing with loading applied along the axis of the femoral tunnel and the bone–patellar tendon–bone graft.

Femoral fixation was performed using two different fixation techniques for ACLR (Figure 1). The femoral ACL footprint was identified, a guide wire was inserted anteromedially, and a 9‐mm cannulated reamer was used to create a femoral tunnel of appropriate length for the respective fixation technique. The proximal bone block was pulled into the tunnel from distally and positioned flush with the joint surface, according to the manufacturers' recommendations. In the CSD group, a fixed‐loop Endobutton CL BTB system (Smith & Nephew) was used for fixation. The continuous loop was passed through a 2‐mm transverse drill hole in the proximal bone block, and the button was advanced through the tunnel. The button was then flipped outside the lateral femoral cortex to achieve cortical anchorage. For the IS group, aperture fixation was achieved using a 7‐mm‐diameter, 20‐mm‐long cannulated titanium IS (Soft Silk; Smith & Nephew), which reflects standard clinical practice for a 9‐mm‐diameter tunnel. The screw was inserted over a guide wire from distally, compressing the bone block against the femoral tunnel wall.

### Biomechanical testing

Biomechanical testing was performed using a servo‐hydraulic materials testing system (MiniBionix 858; MTS Systems) equipped with a 10‐kN load cell (load resolution 1 N; displacement resolution 1 µm). Load and displacement data were recorded at the actuator at 5 Hz using MTS FlexTest 40 software. Specimens were mounted in a custom test jig to allow controlled alignment and uniaxial tensioning. The distal femurs were embedded in cylindrical moulds using polymethylmethacrylate (PALACOS®; Heraeus Medical GmbH), while the distal bone block of the graft was secured to the MTS actuator using a clamp. The test direction was achieved by aligning the graft parallel to the femoral tunnel axis and actuator while constraining all other translations and rotations [3]. The loading protocol was designed to approximate early postoperative rehabilitation as previously reported [3, 7, 14]. After preconditioning with cyclic loading between 10 and 50 N at 0.1 Hz for 10 cycles [14], cyclic displacement testing was performed using sinusoidal loading between 50 and 100 N at 1 Hz until failure or up to 1000 cycles [3, 14]. The protocol was selected to approximate forces encountered during early rehabilitation following ACLR [6]. The loading frequency corresponded to normal walking, as previously described by Honl et al., and the number of cycles approximated the first postoperative week after ACL reconstruction [25]. Specimens that completed cyclic loading without failure were subsequently subjected to quasi‐static tensile testing to failure at a constant displacement rate of 20 mm/min, as previously reported [14]. This failure protocol was chosen to simulate a sudden overload event [25].

#### Outcomes

Outcome measures were derived from the load–displacement curves. These included cyclic displacement between the first and last cycle, ultimate load to failure, and displacement at failure, defined as the maximum load reached before a subsequent load drop on the load–displacement curve. Construct stiffness at failure was calculated based on the corresponding load and displacement values at this point. Failure modes were subsequently inspected visually and categorized for each specimen.

#### Statistics

An a priori sample size calculation was done using the G*POWER software (University of Melbourne). Based on previous published literature, an average standard deviation of 133 N, an alpha of 0.05 and a power of 0.85 revealed that eight specimens were needed in each group to ensure a good study design [9, 14, 18]. Continuous variables are presented as means, and categorical variables as frequencies. Normality of data distributions was assessed. Independent‐samples *t* tests were used to compare mean values between the two femoral fixation groups, with *p* < 0.05 considered statistically significant.

## RESULTS

Eight paired cadaveric specimens from male donors of Caucasian origin with a mean age of 41 years (range: 29–49 years) underwent biomechanical testing. One specimen was omitted from the experiment due to technical errors with the servo‐hydraulic testing machine. Seven specimens completed cyclic loading followed by quasi‐static tensile testing to failure. Descriptive biomechanical data are summarized in Table 1. Overall cyclic displacement across all specimens averaged 2.2 ± 0.9 mm. During quasi‐static testing, the mean ultimate load to failure was 616 ± 173 N, with a mean displacement at failure of 10.1 ± 2.7 mm and a mean stiffness at failure of 65 ± 24 N/mm. Failure modes varied and included suture pull‐out, graft avulsion and bone block fracture. Comparative analyses between fixation methods are presented in Table 2.

**Table 1:** Specimen characteristics and biomechanical outcome measures.

Specimen			Dynamic testing	Quasi‐static testing with outcomes at failure			
Age (years)	Side	Fixation	Displacement (mm)	Displacement (mm)	Stiffness (N/mm)	Load (N)	Failure mode
29	Right	CSD	2.4	10.6	49	520	Lateral fracture
	Left	IS	1.0	9.9	106	1056	Tendon avulsion
39	Right	CSD	2.0	13.3	57	760	Lateral fracture
	Left	IS	1.6	7.0	81	568	Tendon avulsion
41	Left	CSD	1.9	13.2	45	589	Block fracture
	Right	IS	1.5	7.9	110	874	Block fracture
41	Left	CSD	3.5	14.0	39	550	Lateral fracture
	Right	IS	2.5	7.2	63	443	Tendon avulsion
41	Left	CSD	4.5	14.2	28	394	Lateral fracture
	Right	IS	1.9	7.0	76	534	Tendon avulsion
48	Right	CSD	2.5	10.0	54	548	Lateral fracture
	Left	IS	1.5	7.2	82	577	Tendon avulsion
49	Right	CSD	2.6	11.0	53	584	Lateral fracture
	Left	IS	1.7	8.9	71	623	Tendon avulsion
Total (SD)			2.2 (0.9)	10.1 (2.7)	65 (24)	616 (173)	
CSD, mean (SD)			2.8 (0.9)	12.3 (1.7)	46 (10)	563 (109)	
IS, mean (SD)			1.7 (0.5)	7.9 (1.1)	84 (18)	668 (217)	

Abbreviations: CSD, Cortical suspensory device; IS, interference screws; SD, standard deviation.

**Table 2:** Comparison of biomechanical outcomes between fixation methods.

Test	Outcome	Mean difference (CSD‐IS)	95% CI	*p* value
Dynamic	Displacement (mm)	1.1	0.4–1.8	0.008
Quasi‐static	Displacement at failure (mm)	4.4	2.1–6.8	0.004
	Stiffness at failure (N/mm)	−38	−55 to −20	0.002
	Load at failure(N)	−104	−332 to 123	0.31

Abbreviations: CI, confidence interval; CSD, Cortical suspensory device; IS, interference screws.

### Cyclic loading

After cyclic loading, CSD fixation demonstrated greater mean displacement than IS fixation (2.8 ± 0.9 vs. 1.7 ± 0.5 mm), corresponding to a mean difference of 1.1 mm (95% CI, 0.4–1.8; *p* = 0.008).

### Quasi‐static failure testing

During quasi‐static testing, displacement at failure was greater for CSD fixation (12.3 ± 1.7 mm) than for IS fixation (7.9 ± 1.1 mm), corresponding to a mean difference of 4.4 mm (95% CI, 2.1–6.8; *p* = 0.004). Stiffness at failure was lower for CSD fixation (46 ± 10 N/mm) compared with IS fixation (84 ± 18 N/mm; mean difference, −38 N/mm; 95% CI, −55 to −20; *p* = 0.002). No statistically significant difference was observed in ultimate load to failure between fixation methods (563 ± 109 vs. 668 ± 217 N; *p* = 0.31).

### Failure modes

**Figure 2:**
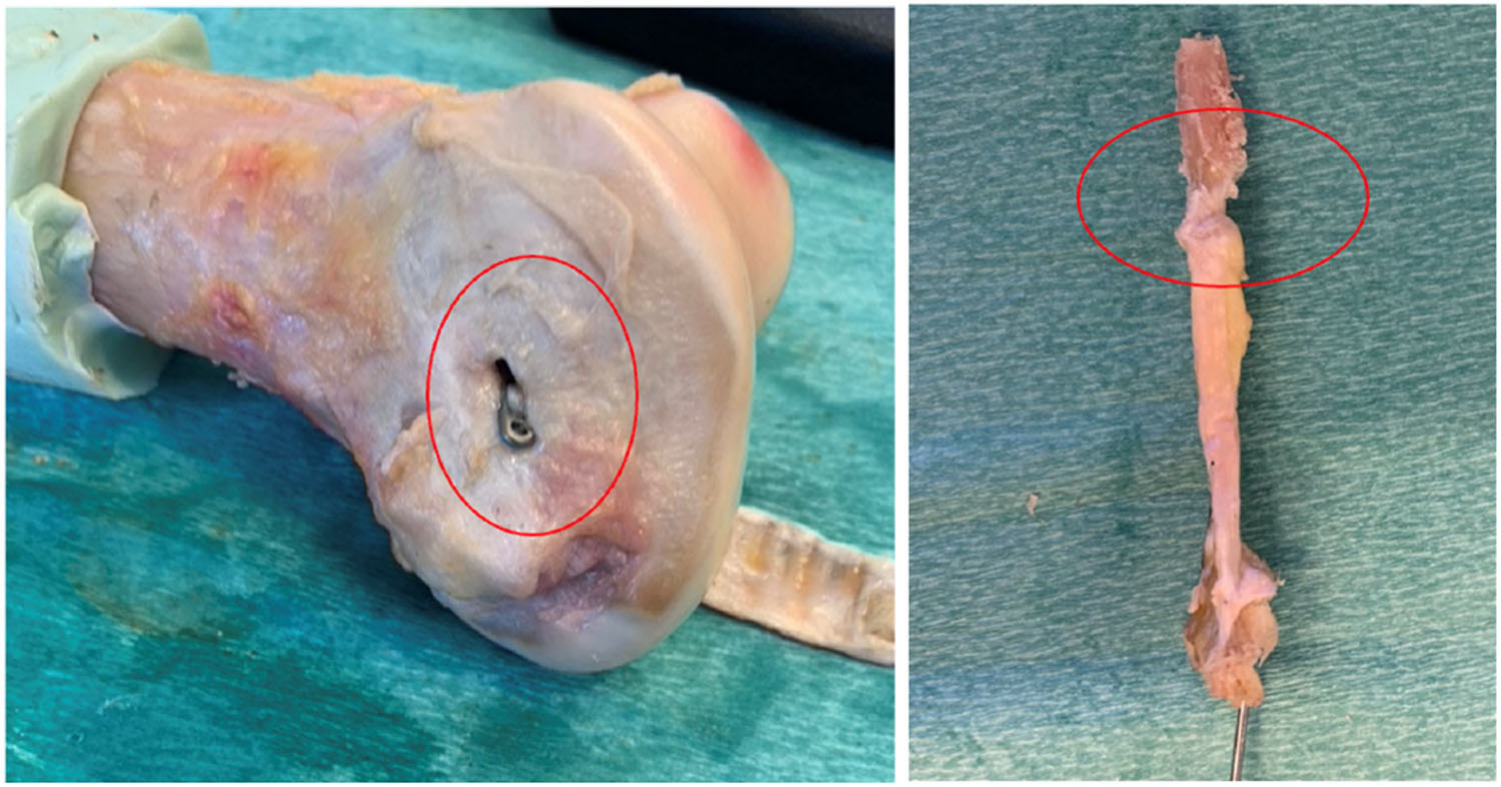
Failure patterns. Left: The pull‐out through the lateral femoral cortex observed with cortical suspensory fixations. Right: The avulsion at the proximal bone–tendon junction observed with interference screw fixations.

Failure most commonly occurred as a pull‐out through the lateral femoral cortex in specimens fixed with a CSD (six of seven specimens), whereas IS fixation predominantly failed by bone–tendon avulsion (six of seven specimens) (Figure 2).

## DISCUSSION

The most important finding of the present study was greater deformation with lower construct stiffness for fixed‐loop CSD compared with IS fixation, whereas ultimate load to failure did not differ despite distinct failure patterns. Under cyclic loading, CSD demonstrated significantly higher construct displacement than IS; however, displacement was below 3 mm which is considered clinically significant displacement in both groups [11].

Regarding cyclic displacement, both CSD and IS fixation measurements in the present study fall within previously reported ranges [3, 7, 14]. The mean displacement was increased with CSD compared to IS fixation during both cyclic and quasi‐static testing, although the magnitude of this difference remained small (Table 1). Displacement with fixed‐loop CSD remains a potential concern. The longer working length with CSD compared with aperture fixation may allow greater elastic deformation, potentially resembling a bungee effect and underscoring the importance of a secure loop design.

The CSD fixation had lower stiffness values compared to IS fixation. Because construct stiffness is largely derived from displacement at failure, the lower CSD stiffness observed appears closely related to displacement behaviour. The observations are consistent with previous studies with hand‐tied CSD designs [3, 7]. The absence of a stiffness difference compared with IS fixation has only been reported after retensioning of adjustable‐loop CSDs [14]. Barrett et al. first described the CSD technique in 1995 [1]. Several biomechanical studies have subsequently evaluated femoral fixation of BPTB grafts [4, 21], although direct comparisons between CSD and IS fixation remain limited (Table 3).

**Table 3:** Overview of previous ex vivo comparisons of CSD and standard IS fixation in femoral BPTB in ACLR.

Variable	Honl et al. [7]	Brown et al. [3]	Mickelson et al. [14]
Type of CSD	Endobutton, Mersilene tape	17 mm plastic button, Ethibond sutures	BTB TightRope
Specimens (group size)	Human cadaveric knees (8)	Human cadaveric knees (5–10)	Porcine femurs, human grafts (8)
Positioning	45° knee flexion	Parallel with bone tunnel	30° knee flexion
Dynamic test	30–300 N, 1 Hz, 60,000 cycles	50–250 N, 1 Hz, 1.000 cycles	50–250 N, 1 Hz, 500 cycles
Quasi‐static test	1 mm/s	1 mm/s	20 mm/min
Mean displacement (mm)	CSD 5 IS: 2 Diff 3*	CSD 4.4 IS 1.5 Diff 2.9*	CSD 2.0 IS 1.1 Diff 0.9*
Failure displacement (mm)	CSD 8 IS 6 Diff 2*	CSD 6.0 IS 3.2 Diff 2.8*	—
Failure stiffness (N/mm)	CSD 107 IS 218 Diff 89*	CSD 207 IS 298 Diff 91*	CSD 220 IS 220 Diff 0
Failure load (N)	CSD 584 IS 837 Diff 253*	CSD 664 IS 710 Diff 46	CSD 700 IS 700 Diff 0
CSD Failure mode	Suture rupture	Suture rupture/pull‐out, block fracture	Block fracture/suture pull‐out

Abbreviations: ACLR, anterior cruciate ligament reconstruction; BPTB, bone–patellar tendon–bone; CI, confidence interval; CSD, Cortical suspensory device; Diff, Difference (maximum value – minimum value); IS, interference screws.

* *p* < 0.05, otherwise statistically non‐significant.

No significant difference in the ultimate loads to failure was detected in the present study, suggesting that CSD may provide solid fixation similar to IS fixation. Comparable ultimate strength between CSD and IS fixation has generally been reported [3, 14]. Lower ultimate failure load with CSD has only been reported when cyclic testing preceded failure testing rather than when failure testing was performed primarily [7]. The lack of difference in ultimate load to failure may reflect a balance between the lateral cortical anchorage and more distal cancellous compression. The maximal ACL force of approximately 400 N reported during rehabilitation exercises [6] suggests that both fixation methods may provide sufficient fixation strength. This interpretation is further supported by the lack of clinical differences in revision risk or quality of life reported in a recent registry study after ACLR, comparing CSD and IS fixation [24].

Distinct failure patterns were observed, with suture pull‐out occurring mainly with CSD in contrast to proximal tendon avulsion with IS fixation. Failure patterns observed with CSD were comparable to those previously described, without identification of new failure patterns [3, 7, 14]. Most likely, failure mechanisms reflect local strain overload at different interfaces (i.e., lateral femoral cortex, bone block fixation point), with failure initiated at the weakest link, indicating different load transfer pathways between CSD and IS fixation. Tendon avulsions observed with IS fixation may also reflect graft damage during insertion. Further, the ultimate differences in failure patterns resulting from overloading may only have clinical relevance under non‐physiological loading conditions.

Taken together, the small but statistically significant difference in displacement and the comparable ultimate strength may indicate adequate fixation. The clinical relevance of the observed displacement difference remains uncertain. However, the significant differences observed despite a limited sample size suggest a distinct mechanical effect of the intervention. They should therefore be considered hypothesis‐generating. As revision rates may not fully reflect the number of patients with less‐than‐optimal outcomes, these cases may possibly be related to subtle construct displacement, which warrants further evaluation, particularly in patients at increased risk of revision ACLR.

The current study has some methodological differences with previous studies which can explain some of the differences observed. Early investigations tested hand‐tied tape or suture‐based constructs [3, 7], whereas later work evaluated contemporary adjustable‐loop designs with manual tensioning and retensioning during testing [14]. In contrast, a fixed‐loop CSD developed specifically for BPTB graft fixation was evaluated here. Human cadaveric specimens were used as previously, although younger than in earlier reports (mean 46 years, range 29–68) [3, 7]. Other methodological differences from the setup include specimen reuse due to limited availability, nonanatomic medial reconstruction with CSD, loading direction at varying degrees of knee flexion, lesser load, but a higher number of loading cycles [3, 7, 14]. However, most displacement has been reported to occur during the first loading cycles [7]. Overall specimen preparation and testing approaches appear comparable with other studies.

## STRENGTHS AND LIMITATIONS

A principal strength of this study lies in the controlled evaluation of both quasi‐static and dynamic mechanics prior to failure, which underlies the findings of displacement and strength. Due to the limited availability of young human cadaveric specimens, somewhat older specimens were included. However, specimens with pathology indicating reduced bone mineral density were not included to improve extrapolation to younger adults. Otherwise, the randomized paired design enabled intrapair comparisons independent of bone mineral variability. This design strengthens interpretation of relative differences, while absolute values that differed somewhat from previous reports may be setup‐related, such as by the worst‐case load direction. Limitations must nevertheless be acknowledged. Despite efforts to ensure clinically relevant testing conditions, the absence of intact soft‐tissue coverage and in vivo biological responses makes the study exploratory. Importantly, graft–bone motion was not directly quantified, as no extensometer or video‐based tracking was performed. Interpretation therefore relies partly on extrapolation from previous studies suggesting that cyclic displacement may reflect graft–tunnel motion [3]. Regarding external validity, the present findings may be limited to femoral fixation, as different biomechanical factors may influence fixation on the tibial side. Furthermore, the results may be specific to the implants and dimensions tested. The lack of bone mineral density testing is a limitation despite the above‐mentioned efforts to include specimens without pathological bone mineral density. The loading values (50–100 N) in the present study are relatively low compared to other studies and may underestimate the construct displacement under higher physiological loads. Finally, one specimen was omitted from the cyclic testing due to technical errors, and therefore the study may be underpowered with a sample size of seven specimens.

## CONCLUSION

Fixed‐loop CSD fixation of BPTB grafts demonstrated greater deformation and lower stiffness than IS fixation, and with similar ultimate failure strength. The clinical relevance of the observed difference remains uncertain.

## AUTHOR CONTRIBUTIONS

All authors contributed to the study conception and design. Material preparation, data collection and analysis were performed by Alexander Nagel Tandberg, Marek Zegzdryn and Jan‐Egil Brattgjerd. The first draft of the manuscript was written by Alexander Nagel Tandberg, and all authors commented on previous versions of the manuscript. All authors read and approved the final manuscript.

## CONFLICT OF INTEREST STATEMENT

The authors declare no conflicts of interest.

## ETHICS STATEMENT

This study was approved by the Regional Committee for Medical and Health Research Ethics (approval no. 2018/2182).

## Data Availability

The data that support the findings of this study are available from the corresponding author upon reasonable request.
